# In-Work Poverty in Times of COVID-19

**DOI:** 10.1007/978-3-030-65355-2_5

**Published:** 2021-03-20

**Authors:** Sonja Bekker, Johanna Buerkert, Quirine Quirijns, Ioana Pop

**Affiliations:** 1 Tilburg University, Tilburg, The Netherlands; 2 Tilburg University, Tilburg, The Netherlands; 3 Tilburg University, Tilburg, The Netherlands; 4 Tilburg University, Tilburg, The Netherlands; 5 Research Cluster Empirical Research into Institutions for conflict resolution, Utrecht University School of Law, Utrecht, The Netherlands; 6 Center for Water Oceans and Sustainability, Law Utrecht University, Tilburg, The Netherlands; 7 Tilburg Law School, Tilburg, The Netherlands; 8 Department of Sociology, Tilburg School of Social and Behavioral Sciences, Tilburg, The Netherlands

## Abstract

The corona crisis has an unequal impact on worker’s income. Workers with unstable jobs prior to the crisis, have been affected hardest due to the loss of work and income (Börner, 2020). An example is the group of workers who cannot make ends meet, despite having a job. In order to explore the impact of the coronavirus crisis on in-work poverty, it is relevant to get a better insight into how low income is defined because in the Netherlands low income and poverty are calculated in various ways. For this chapter we use two indicators (Statistics Netherlands, 2018; SCP, 2018). The first is the poverty threshold, indicating whether or not the income is sufficient to meet basic needs such as buying food, housing, and participating in social activities. The second is the low-income threshold, representing stable purchasing power over time.

The corona crisis has an unequal impact on worker’s income. Workers with unstable jobs prior to the crisis, have been affected hardest due to the loss of work and income (Börner 2020). An example is the group of workers who cannot make ends meet, despite having a job. In order to explore the impact of the coronavirus crisis on in-work poverty, it is relevant to get a better insight into how low income is defined because in the Netherlands low income and poverty are calculated in various ways. For this chapter, we use two indicators (Statistics Netherlands 2018a; SCP 2018). The first is the poverty threshold, indicating whether or not the income is sufficient to meet basic needs such as buying food, housing, and participating in social activities. This amounted to €1135 per month for a single person household in 2017. The second is the low-income threshold, representing stable purchasing power over time, which amounted to €1060 per month for a single person household in 2018 (Statistics Netherlands 2018b, 2019a; SCP 2018). Data shows that 7.9% of Dutch households earned below this low-income threshold. Within the group of workers, 2.4% was part of a household earning below the low-income threshold. This means that having an income from work (instead of from social security) decreases the likelihood of having a very low income (Statistics Netherlands 2019b). Using the poverty threshold, in 2017, 5.7% of the Dutch population lived in a household experiencing poverty. From this perspective, being employed also decreases the likelihood of being poor, with only 2% of employees compared to 8% of self-employed having an income below the poverty threshold (SCP 2020).

Overall, in-work poverty in the Netherlands is quite low, especially compared to the other 28 EU countries (Eurostat 2020). As a result, the problem is not always seen as significant. Still, in numbers, 125,000 employees and 95,000 self-employed lived in a poor household in 2017. Moreover, some groups of workers have a much higher chance to experience in-work poverty. In general, two factors contribute to in-work poverty: a low hourly income and a low number of working hours per week (SCP 2018, 2020).

## Flexible Workers Have a Higher Chance at In-Work Poverty

Regarding the poverty threshold, the data show that the solo self-employed people (12.6%), on-call workers (10.2%), self-employed people with personnel (8.3%), people with small part-time jobs (6.5%), and temporary agency workers (8%) have a much higher risk at in-work poverty or social exclusion than employees with a full-time open-ended employment contract (0.7%) (data 2014) (SCP 2018). Similarly, being covered by a collective labor agreement lowers the likelihood of in-work poverty.

Young people, particularly those working in the retail and hospitality sectors, are more likely to experience in-work poverty (Van Deurzen et al. 2018). Most workers in these two sectors are under the age of 30 (in retail even under the age of 25) and often only employed part-time (Statistics Netherlands 2018c; WerkNL 2018). Due to the layered Minimum Wage System in the Netherlands (which includes lower minimum wages for youth aged below 21), young people have quite low incomes even if they work full time. For instance, a 19-year-old earning at minimum wage level in 2020 would have a gross income of €1008 per month, which is below the low-income threshold. The solo self-employed workers are especially vulnerable to the changing economy as they largely fall outside of the Dutch social security system. Besides income insecurities, this group is at a higher risk when they become ill or reach the pensionable age (Statistics Netherlands 2018c).

An important vulnerability for young people and flexible workers is that they often are the first ones to become redundant during economic crises while having built little entitlements to social security. The next section explores whether the groups mentioned in this section are indeed affected the most due to the coronavirus crisis.

## Effects of the Crisis on the Income of Vulnerable Groups

The immediate effects of the coronavirus crisis on the loss of jobs and income have not been translated into current statistics yet. However, first explorations suggest that the most vulnerable groups on the labor market have been affected the most. In March 2020, the overall number of jobs decreased by 23,000, with large job losses in the hospitality and financial services sectors although in education and health care the number of jobs is still growing (Statistics Netherlands 2020a).

Especially “flexible” jobs disappeared in March 2020: on-call jobs (–65,000), temporary agency jobs (–8000), and fixed-term jobs (–7000). Notably, people with flexible jobs in professions that could not be carried out due to the lockdown have lost their employment. Examples are sales employees in retail and waiters in bars and restaurants (Statistics Netherlands 2020b). Regarding self-employed workers, the coronavirus impact on their work may be sketched by the use of the Temporary Subsidy for Self-Employed (Tozo: Tijdelijke overbruggingsregeling zelfstandig ondernemers). Tozo has been designed by the government specially to give income support to self-employed people who lost their income due to the coronavirus crisis. By April 30, about 343,000 Tozo applications were submitted (FNV 2020). This shows that the need for it is high. In May 2020, the organization Wijzer in geldzaken also looked at income loss due to the coronavirus (Wijzer in geldzaken 2020). Based on a sample of 1219 respondents (A population representative sample of 532 combined with a sample of 687 participants from six financially vulnerable groups), the results emphasize that the effects of the coronavirus crisis are different across groups. For instance, low-income employees with a permanent contract report either lower (22%) or more (8%) working hours. In contrast, low-income employees with a flexible contract say that they receive fewer assignments (41%) and that they work fewer hours (19%). Those who are self-employed report that they receive fewer assignments (40% of those with low incomes and 31% of those with high incomes).

This situation is reflected in the financial situation of these workers, with several groups reporting a stronger decrease of their income, in comparison to other groups of workers, i.e., those under payment employment: 62% of the low-income employees with flexible contracts, 69% of the low-income self-employed workers, and 49% of the high-income self-employed workers. It seems that the groups who already struggled to make ends meet prior to the crisis are often also the groups who are hit hardest by the loss of employment and income due to COVID-19.

## Conclusion

Now that the COVID-19 pandemic is turning from a health crisis into an economic and social crisis, the flaws in the labor market and social security have become acutely visible. In particular, the groups of workers who already had a vulnerable position on the labor market have been hit hardest by the first shocks of the crisis. This includes workers with unstable jobs and a low income prior to the crisis, such as low-income workers with flexible jobs and solo self-employed workers.

This links back to some of the main failures of the “old common”: persisting inequalities and a generational divide. Young people and “flex workers” are amongst those suffering most from the current crisis in terms of financial consequences. Therefore, the crisis exacerbates the already existing weaknesses of the old common. In relations to the New Common, this means that those outside the traditional standard employment contracts must not be forgotten when reforming the labor market. Especially in the light of ongoing digital transformation, ways to secure the livelihood of all workers need to be found. The current findings show that even in modern labor markets, workers with a standard employment contract are protected best: full-time employed workers with an open-ended contract and covered by a collective labor agreement. This seemed already valid prior to the crisis and has become more urgently visible within this first phase of the pandemic. Deviating from the full-time and open-ended contract standard is a risky affair, especially for those who already have a low income to begin with. Moving towards a new and more inclusive common would thus entail an encompassing labor market reform, ensuring that all workers—irrespective of their employment relationship, age, or profession—have an income that meets basic needs, both within and after the crisis.
